# Mechanisms Underlying Latent Disease Risk Associated with Early-Life Arsenic Exposure: Current Research Trends and Scientific Gaps

**DOI:** 10.1289/ehp.1409360

**Published:** 2015-06-26

**Authors:** Kathryn A. Bailey, Allan H. Smith, Erik J. Tokar, Joseph H. Graziano, Kyoung-Woong Kim, Panida Navasumrit, Mathuros Ruchirawat, Apinya Thiantanawat, William A. Suk, Rebecca C. Fry

**Affiliations:** 1 Department of Environmental Sciences and Engineering, UNC Gillings School of Global Public Health, Chapel Hill, North Carolina, USA; 2 Arsenic Health Effects Research Program, School of Public Health, University of California, Berkeley, Berkeley, California, USA; 3 National Toxicology Program, National Institute of Environmental Health Sciences (NIEHS), National Institutes of Health (NIH), Department of Health and Human Services (DHHS), Research Triangle Park, North Carolina, USA; 4 Department of Environmental Health Sciences, Columbia University Mailman School of Public Health, New York, New York, USA; 5 School of Environmental Science and Engineering, Gwangju Institute of Science and Technology (GIST), Gwangju, Republic of Korea; 6 Laboratory of Environmental Toxicology, Chulabhorn Research Institute, Bangkok, Thailand; 7 Superfund Research Program, NIEHS, NIH, DHHS, Research Triangle Park, North Carolina, USA

## Abstract

**Background:**

Millions of individuals worldwide, particularly those living in rural and developing areas, are exposed to harmful levels of inorganic arsenic (iAs) in their drinking water. Inorganic As exposure during key developmental periods is associated with a variety of adverse health effects, including those that are evident in adulthood. There is considerable interest in identifying the molecular mechanisms that relate early-life iAs exposure to the development of these latent diseases, particularly in relationship to cancer.

**Objectives:**

This work summarizes research on the molecular mechanisms that underlie the increased risk of cancer development in adulthood that is associated with early-life iAs exposure.

**Discussion:**

Epigenetic reprogramming that imparts functional changes in gene expression, the development of cancer stem cells, and immunomodulation are plausible underlying mechanisms by which early-life iAs exposure elicits latent carcinogenic effects.

**Conclusions:**

Evidence is mounting that relates early-life iAs exposure and cancer development later in life. Future research should include animal studies that address mechanistic hypotheses and studies of human populations that integrate early-life exposure, molecular alterations, and latent disease outcomes.

**Citation:**

Bailey KA, Smith AH, Tokar EJ, Graziano JH, Kim KW, Navasumrit P, Ruchirawat M, Thiantanawat A, Suk WA, Fry RC. 2016. Mechanisms underlying latent disease risk associated with early-life arsenic exposure: current research trends and scientific gaps. Environ Health Perspect 124:170–175; http://dx.doi.org/10.1289/ehp.1409360

## Introduction

It is estimated that 10% of the worldwide disease burden is attributable to inadequate standards in drinking water, sanitation, and hygiene (Prüss-Üstün et al. 2008). One of the largest threats to human health worldwide, particularly in rural and developing areas, is the presence of high levels of inorganic arsenic (iAs) in drinking water. Although the World Health Organization (WHO) has recommended that the levels of iAs in drinking water should not exceed 10 ppb (WHO 2011), > 100 million people worldwide are believed to be exposed to levels in drinking water that exceed this limit (Uddin and Huda 2011). Much of this exposure is attributable to the use of iAs-contaminated groundwater as a source of drinking water. Although groundwater may be contaminated with iAs due to anthropogenic activities, it is most often contaminated due to geological leaching (Garelick et al. 2008). Rural areas and developing countries are often particularly impacted based on their reliance on groundwater as a source of drinking water. In terms of affected populations, the impact of this exposure is perhaps most evident in areas of Southeast Asia, including Bangladesh and India (Mukherjee et al. 2006; Sen and Biswas 2013). For instance, it has been estimated that 35–77 million of the 125 million inhabitants of Bangladesh may have been exposed to iAs levels in drinking water that exceed the WHO standard (Karagas 2010; Smith et al. 2000). In addition to the situation in Southeast Asia, it is becoming increasingly clear that iAs contamination of groundwater and surface waters is a worldwide problem. Inorganic As concentrations in groundwater have been detected around the globe at levels that greatly exceed the WHO standard, suggesting many more populations may be at risk (Ravenscroft et al. 2009). Although countries such as the United States have adopted the WHO standard for limiting iAs exposure via municipally supplied water, many people in these countries, perhaps numbering in the millions, may still be exposed to relatively high levels of iAs via use of unregulated private wells supplied with groundwater (George et al. 2006; Sanders et al. 2012). Exposure for populations who generally have more moderate iAs exposure from drinking water than Southeast Asian populations also may occur through the consumption of certain foods such as rice and rice products [Cottingham et al. 2013; Davis et al. 2012; European Food Safety Authority (EFSA) 2009]. This is of particular concern to vulnerable subpopulations such as infants and young children who have increased susceptibility to toxicants and may consume relatively large proportions of rice-containing products compared with adults.

Numerous and varied health effects are attributed to iAs exposure in human populations, such as adverse pregnancy/birth outcomes, increased morbidity and mortality in infants and children, cognitive impairments in children, and the development of noncommunicable diseases in adults including cardiovascular disease, peripheral vascular disease, chronic respiratory disease, and various cancers (Mazumder et al. 2005; Parvez et al. 2013; Rahman A et al. 2007, 2011; Rahman MM et al. 2009; Raqib et al. 2009; Smith and Steinmaus 2009; Wasserman et al. 2004, 2007). The carcinogenic impacts of chronic iAs exposure are among the best-characterized and most intensely studied effects of iAs. These effects have been reported at a wide variety of iAs concentrations including relatively low concentrations observed in the United States (Garcia-Esquinas et al. 2013; Gilbert-Diamond et al. 2013). Inorganic As is classified as a known human carcinogen that targets multiple organs including the skin, lung, urinary bladder, and liver (International Agency for Research on Cancer 2012; National Research Council 2001). Elucidating the molecular mechanisms that link iAs exposure to disease has proven to be a daunting task given the complexity of disease states and multiplicity of factors that likely influence disease development. Several mechanisms have been implicated in the development of iAs-associated cancers, including the generation of oxidative stress, interference with DNA repair, alterations in cellular signaling, and alterations to the genome and epigenome. No single mechanism has emerged as a key event, and it is likely that iAs exerts carcinogenic effects via multiple mechanisms.

In the present commentary we summarize the current state of knowledge of the relationship between early-life iAs exposure and altered disease risk. In particular, we highlight research needs that would best elucidate the molecular mechanisms linking early-life exposure to iAs and cancer development later in life. Epigenetic reprogramming has been a major research focus in this area, and we highlight this as well as two additional and likely interacting mechanisms that may contribute to carcinogenic effects associated with early-life iAs exposure, namely the development of cancer stem cells (CSCs) and perturbed immune function.

## Early-Life Inorganic Arsenic Exposure: Evidence for the Development of Cancers in Animals Models and Human Populations

In both exposed human populations and experimental animals, exposure to iAs during critical developmental periods has been shown to be sufficient to alter the risk of developing adverse health effects much later in life (reviewed by Farzan et al. 2013a). For instance, iAs has been shown to cause delayed carcinogenic effects in several strains of mice when they are exposed briefly during the prenatal period. This effect was first observed in C3H mice, in which offspring born to pregnant females exposed briefly to high doses of sodium arsenite [NaAs_2_O_3;_ iAs(III)] in drinking water [42.5 or 85 ppm arsenic during gestational days (GD) 8–18] resulted in increased incidence and/or multiplicity of tumors in multiple organs in both female and male adult offspring (Waalkes et al. 2003, 2004b). Sex-specific effects were observed, including a pronounced dose–response effect in the incidence and multiplicity of hepatocellular carcinoma (HCC), a major form of cancer associated with chronic iAs exposure in humans, in male offspring (Waalkes et al. 2003). Exposure to iAs(III) prenatally as described above followed by postnatal application of tumor-promoting agents was also found to produce cancers, tumors, and/or proliferative lesions in some organs, and to have initiating effects in other organs in several strains of mice (Tokar et al. 2010a; Waalkes et al. 2004c, 2006a, 2006b, 2008). Prenatal iAs exposure is also associated with accelerated/exacerbated atherosclerosis in apolipoprotein A-knockout (ApoE^–^/^–^) mice, a mouse strain used to study atherosclerotic disease. In this model, male offspring born to pregnant ApoE^–^/^–^ mice administered 85 ppm iAs(III) from GD8 to birth (GD20) is associated with evidence of increased atherosclerotic disease in male offspring 10 and 16 weeks after birth compared to controls (Srivastava et al. 2007). Other effects have also been reported at significantly lower doses, such as cognitive impairments in adult C57B16/J mice that were born to dams exposed to 100 μg/L iAs in drinking water from 1 week before conception until birth (Cronican et al. 2013).

In epidemiologic studies with an ecological design, a defined period of iAs exposure in northern Chile revealed several similarities to the mouse studies cited above, namely that early-life exposure to iAs was associated with a variety of latent health impacts, some of which are sex dependent. For instance, young adults (age 30–49 years) who experienced prenatal and/or childhood exposure via high levels of iAs in municipally supplied drinking water (average level, 860 μg/L) in the Antofagasta region of northern Chile between 1958 and 1970 had increased mortality from both cancerous and noncancerous effects, including chronic renal disease, chronic lung disease, acute myocardial infarction, and cancers of the urinary bladder, larynx, kidney, lung, and liver (Smith et al. 2006, 2012; Steinmaus et al. 2014; Yuan et al. 2007, 2010). Although ecologic studies may be limited by a lack of individual exposure and outcome data, these findings have been supported by a case–control study in the same part of Chile with individual data linking the early life exposures with increased cancer risk in adults (Steinmaus et al. 2014). Taken together, these results highlight the sensitivity of early-life periods to the effects of iAs, notably at very high levels of exposure, and a major research focus in recent years has been to understand the molecular alterations that link exposure during these periods to delayed effects, particularly cancers, later in life.

As discussed in more detail below, analyses of tissue from target organs in C3H and ApoE^–^/^–^ mice that were prenatally exposed to iAs indicated that this altered disease susceptibility was associated with persistent changes in the expression and epigenetic landscape of genes implicated in disease development (Liu et al. 2004; States et al. 2012; Waalkes et al. 2004a; Xie et al. 2007). These observations are consistent with theories that suggest permanent alterations or “reprogramming” of the epigenome is a critical link between toxicant exposure in early life and altered disease risk later in life (Bollati and Baccarelli 2010; Jirtle and Skinner 2007). The epigenome, widely considered to include DNA methylation, histone post-translational modifications, and microRNAs (miRNAs), plays a critical role in the regulation of gene expression; therefore, epigenetic reprogramming by toxicants may alter the susceptibility to adverse health outcomes by causing persistent changes in expression patterns of genes involved in disease development. Epigenetic reprogramming has been a major focus of research efforts that have set out to elucidate the molecular mechanisms that underlie the link between early-life iAs exposure and altered disease risk.

## Molecular Mechanisms Underlying Latent Carcinogenic Effects Associated with Early-Life Arsenic Exposure

*Epigenetic reprogramming/alterations in gene expression.* There are several indications in transplacental exposure models that iAs may alter disease risk in adulthood via epigenetic reprograming and altered gene expression. For instance, liver tissue isolated from newborn male mice exposed to hepatocarcinogenic doses of iAs *in utero* had alterations in GC-rich regions throughout the genome and altered expression of genes implicated in liver tumorigenesis (Waalkes et al. 2004a; Xie et al. 2007). The expression profiles of similar disease-associated genes were also observed in HCCs and peritumorous tissue in transplacentally exposed adult male mice (Liu et al. 2004; Waalkes et al. 2004a; Xie et al. 2007). Of particular interest was the increased expression of estrogen receptor alpha (*Er-*α), which was concurrent with hypomethylation of several regions of DNA in the *Er-*α promoter (Waalkes et al. 2004a). This is of potential significance because there is often an inverse relationship between promoter methylation status and transcriptional competency. In the ApoE^–^/^–^ atherosclerosis model, brief prenatal exposure to 49 ppm iAs(III) in maternal drinking water was shown to alter the transcriptional profiles in the livers of male mice that were evident at postnatal day (PND) 1 and PND 70; interestingly, the altered mRNA and miRNA profiles differed between these two time points, which is consistent with reprogramming of gene expression profiles in the liver (States et al. 2012). Although the mouse transplacental carcinogenesis models support an associative relationship between alterations in DNA methylation and the altered expression of key genes, it is unknown whether this relationship is causal, and the mechanisms that underlie the altered DNA methylation patterns are unknown. The molecular events that cause the reprogramming of gene expression in the mouse atherosclerosis model are also unknown and must be explored. In addition, the impact of other epigenetic alterations in the developmental origins of iAs-based disease has been relatively unexplored in animal models. One study revealed an association in C57B16/J mice between transplacental exposure to 100 μg/L iAs in maternal drinking water, alterations in global H3K9 acetylation patterns, and cognitive impairments in adulthood, which is of potential significance because changes in histone acetylation patterns have been reported in cognitive insufficiencies (Cronican et al. 2013). These studies also highlight that epigenetic alterations may be a mechanism by which iAs may cause a variety of adverse health effects throughout the lifetime of an individual, many of which are likely unrecognized. Chronic respiratory symptoms have been reported to be increased in children following early-life exposure to iAs in Bangladesh, although without evidence of reduced lung function (Smith et al. 2013). A recent study reported that early-life exposure to iAs was associated with reduced lung function in children (Recio-Vega et al. 2015). The results of these studies need to be confirmed, but they underscore that long latency periods are not necessarily associated with all adverse health effects associated with early-life iAs exposure.

A major area of focus in recent years has been the examination of alterations in gene expression and epigenetic alterations, including DNA methylation and miRNAs, that are associated with early-life iAs exposure in humans. Many of these analyses have focused on alterations observed in cord blood leukocytes. Related to prenatal iAs exposure and transcriptional change, a transcriptomics approach identified 447 iAs-responsive genes in cord blood leukocytes in newborns from Thailand (*n* = 32; range of maternal toenail arsenic levels in unexposed subjects, 0.10–0.38 μg/g; or exposed subjects, 0.50–68.63 μg/g) (Fry et al. 2007). Most of these genes had increased expression levels associated with iAs exposure and were enriched for roles in immune and inflammatory response, stress response, and cancer/tumorigenesis. Related to epigenetic regulation of the transcriptional response, in Mexican newborns (*n* = 40; range of iAs in maternal drinking water = < 1 to 236 μg/L), 12 miRNAs and 334 mRNAs were identified that were correlated with arsenic levels of total urinary arsenic in maternal urine (range of U-tAs, 6.2–319.7 μg/L) (Rager et al. 2014). Functional analyses indicated the modulated miRNAs are known to be regulators of cellular responses such as inflammation and diseases with known links to iAs exposure including cancer and diabetes mellitus. Nine of the modulated miRNAs were predicted to regulate the expression levels of approximately 20% of the U-tAs-associated mRNAs, providing an indication that miRNAs regulate, at least to some extent, the transcriptional response to prenatal iAs exposure.

Several analyses have examined iAs-associated perturbations of cord blood proteins. For instance, the level of several proinflammatory cytokines in the cord blood of Bangladeshi newborns (*n* = 130; median U-tAs = 66 μg/L), namely interleukin 1 beta (IL-1β), interleukin 8 (IL-8), tumor necrosis factor (TNF), and interferon gamma (INF-γ), had U-shaped curves across quartiles of maternal U-tAs at gestational week 30 (GW30) (Ahmed et al. 2011). Using a proteomic strategy, Bailey et al. (2014) identified a total of 111 maternal U-tAs–associated cord blood proteins in Mexican newborns (*n* = 50; range of U-tAs, 6.2–319.7 μg/L). These proteins were enriched in functions related to immune/inflammatory response and cellular development/proliferation, and the majority were predicted to be regulated by TNF, a protein that is implicated in the development of several adverse health effects associated with iAs exposure. Most newborns had a positive association between the levels of proteins and U-tAs, which was consistent with a proinflammatory response. However, this relationship was inverted in some newborns, underscoring an interindividual response to prenatal iAs exposure that may impact susceptibility to adverse health outcomes. The basis for these interindividual differences remains unknown but may be related to iAs metabolism. These studies reveal that at the mRNA, miRNA, and protein levels, genes with similar functions such as immune response are altered in the cord blood of prenatally exposed newborns across several populations. As discussed below, alterations in immune function are implicated in a variety of adverse health impacts associated with early-life iAs exposure. Still, a precise mechanistic relationship between these alterations and adverse health impacts remains unknown. Furthermore, although leukocytes have roles in immune function, the relevance of altered gene expression profiles in leukocytes to the development/susceptibility of adverse effects in other target tissues is unclear.

Studies that have examined epigenetic effects associated with early-life iAs exposure have focused on changes in DNA methylation at both the gene-specific level and global level (Intarasunanont et al. 2012; Kile et al. 2012; Pilsner et al. 2012). Gene-specific changes may be significant because they impart functional changes in gene expression levels. Global changes in methylation status of the genome may also be important because such changes may be associated with genetic instability. More recent studies have used genome-wide approaches to identify associations between iAs exposure and the methylation status of individual CpG sites, the primary target of DNA methylation in humans (Broberg et al. 2014; Kile et al. 2014; Koestler et al. 2013; Rojas et al. 2015).

Sex-dependent relationships have been observed between the global DNA methylation status of cord blood leukocytes of Bangladeshi newborns (*n* = 101; range of iAs in maternal drinking water, 0.01–66.1 μg/L), and prenatal iAs exposure, in which this relationship was positive in newborn males and negative in newborn females (Pilsner et al. 2012). In Thailand (*n* = 71; range of iAs in maternal drinking water, 0.18–8.38 μg/L), the methylation status of long interspersed element 1 (LINE-1) elements was used as an indicator of changes in global DNA methylation levels (Intarasunanont et al. 2012). No associations between LINE-1 methylation and indicators of prenatal iAs exposure were observed, but this study did identify a positive relationship between *in utero* arsenic exposure and DNA methylation of the tumor protein 53 (*TP53*) promoter. In iAs-exposed mother–child pairs in Bangladesh (*n* = 113; range of iAs in maternal drinking water, < 1 to 230 μg/L), the relationship between iAs exposure and DNA methylation patterns of LINE-1 and the *TP53* and cyclin-dependent kinase inhibitor 2A (*CDKN2A*/*p16*) promoters were examined in maternal and umbilical cord leukocytes (Kile et al. 2012). Similar DNA methylation patterns were observed in mothers and newborns, which included a positive association between maternal U-tAs and LINE-1 methylation and some CpG sites within the *p16* promoter. Evidence of a nonlinear dose–response relationship was also observed, as the most effects were observed in the middle tertiles of exposure. These results raise the possibility that epigenetic effects may be observed at relatively moderate levels of exposure, and that dose–response relationships may be complex, thus underscoring the need for follow-up studies.

Several genome-wide analyses have identified changes in cord blood DNA methylation profiles associated with prenatal iAs exposure. One study identified changes observed in New Hampshire newborns exposed to relatively low levels of iAs *in utero* (average iAs in maternal drinking water, 1.2 μg/L; average U-tAs, 4.1 μg/L) (Koestler et al. 2013). Several DNA regions were identified in which there was a relationship between DNA methylation levels and maternal U-tAs, but no overall change in the global DNA methylation status was observed. Most of these differentially methylated regions were found to be within CpG islands, the CpG-rich areas of euchromatin known to epigenetically regulate gene expression (Koestler et al. 2013). In Bangladesh (*n* = 127; median maternal U-tAs at GW30, 89 μg/L), an inverse correlation was observed between maternal U-tAs and the DNA methylation status of cord blood CpG sites predominantly in boys, and pathway-level analyses of differentially methylated genes revealed an overrepresentation of functions associated with cancer development in boys compared with girls (Broberg et al. 2014). Another genome-wide analysis of cord blood of prenatally exposed Bangladeshi newborns identified differential methylation within CpG regions of cord blood leukocytes in relationship to prenatal arsenic exposure. Importantly, this analysis identified a positive association of DNA methylation of CpG regions within 71 genes and levels with iAs in maternal drinking water after adjusting for U-tAs–associated shifts in leukocyte populations, thus highlighting that alterations in DNA methylation profiles must be corrected for shifts in leukocyte populations that may occur due to iAs exposure (Kile et al. 2014). To date, one genome-wide study has investigated the relationship between changes in DNA methylation and gene expression in prenatally exposed newborns (Rojas et al. 2015). In this Mexican population (*n* = 38; arsenic in maternal drinking water, 1–236 μg/L; mean U-tAs, 73.87 μg/L), the methylation status of CpG sites from 2,919 genes isolated from newborn cord blood was found to be associated with prenatal iAs exposure. DNA methylation changes within the first exon and within 200 bp of the transcription start site yielded the most significant association with gene expression profiles. In addition, a set of 7 target genes including growth-associated and diabetes-associated genes such as the imprinted gene *KCNQ1* (potassium voltage–gated channel, KQT-like subfamily, member 1) were differentially methylated, differentially expressed, and associated with birth outcomes/measures in relationship to prenatal arsenic exposure (Rojas et al. 2015). This research identified a commonality at the sequence level of the differentially methylated genes related to transcriptional factor binding. This transcription factor occupancy was proposed as a basis for gene-specific DNA methylation patterning that could underlie responses to iAs and other environmental contaminants (Sanders et al. 2014).

Taken together, these studies reveal several important findings: that iAs-associated alterations in DNA methylation patterns are observed across populations that are exposed to varying levels of iAs, and that complex dose–response relationships have been observed. Effects may be sex-dependent and mother–child pairs have similar alteration patterns, which may have relevance to sex-dependent and interindividual susceptibility to iAs-associated disease. The relevance of these alterations to long-term disease susceptibility and, to a large extent, changes in gene expression patterns at the mRNA or protein level remains unknown and must be explored. As with gene expression patterns, the relevance of changes in DNA methylation patterns in blood leukocytes to disease risk in various target organs is also unknown.

*Cancer stem cells. In vitro* and *in vivo* evidence has indicated that the emergence of CSCs after prenatal iAs exposure may play important roles in the development of some iAs-associated cancers, including those associated with prenatal iAs exposure. CSCs have altered stem cell (SC) functions but retain some characteristics that are key for their survival and later development into cancers such as self-renewal, quiescence, ability to differentiate, and conditional mortality (Tokar et al. 2011). In the iAs-exposed fetus, CSCs are believed to arise from normal SCs or slightly differentiated SC progeny and remain quiescent in target organs until ultimately transforming into malignant cells (Tokar et al. 2011). Interestingly, evidence suggests that CSC overabundance is a characteristic not of all carcinogens but of at least some iAs-associated cancers; for instance, the malignant transformation of the prostate epithelial cell line RWPE-1 by iAs exposure *in vitro* resulted in an overabundance of CSCs compared with RWPE-1 cells transformed by cadmium or *N*-methyl-*N*-nitrosurea (Tokar et al. 2010b). Arsenic-transformed malignant epithelial cells have also been shown to “recruit” normal SCs into a CSC phenotype (Xu et al. 2012), which may help explain the CSC overabundance often observed in iAs-induced tumors and iAs-transformed cell lines (Tokar et al. 2010b).

The triggers that end the quiescent state to produce cancers are unknown but may include the effects of continued exposure to iAs during the postnatal period or postnatal exposure to other chemicals. For instance, an overabundance of CSCs was observed in the highly aggressive squamous-cell carcinomas (SSCs) that developed in Tg.AC mice after prenatally exposure to carcinogenic doses (42.5 and 85 ppm) of iAs(III) followed by postnatal application of 2 μg/0.1 mL acetone 12-O-tetradecanoyl phorbol-13-acetate to the skin for 36 weeks (Waalkes et al. 2008). There is evidence that aberrant gene expression is a key, early event in the development of CSCs. For instance, the fetal skin of Tg.AC mice that were prenatally exposed to iAs demonstrated increased expression of several genes associated with SC/carcinogenesis functions including the oncogene *v-Ha-ras*, a cell surface marker of keratinocyte SCs and cancer skin cells (CD34), and a gene involved in keratinocyte SC renewal, namely ras-related C3 botulinum toxin substrate 1 (*Rac1*). Similar to results observed in fetal skin, an examination of the highly aggressive SCCs that developed in Tg.AC mice after prenatal arsenic exposure and postnatal TPA exposure also revealed high expression of *v-Ha-ras* and the CSC markers *CD34* and *Rac* compared with tumors that arose after TPA treatment alone (Waalkes et al. 2008). A comparison of the tumors arising from these two treatment groups showed increased expression levels of the pro-growth gene *Cd1* and decreased expression of tumor suppressor *p16* in the aggressive SCCs in the iAs arsenic/TPA group (Waalkes et al. 2008). These data demonstrate a relationship between gene expression and the acquisition of malignant phenotype. The mechanisms that link iAs exposure and changes in gene expression patterns of these key genes in CSCs remain understudied and must be addressed. No alterations were shown in the DNA methylation status of the *v-Ha-ras* promoter concurrent with increased *v-Ha-ras* expression (Waalkes et al. 2008), even though *v-Ha-ras* expression has been shown to be controlled by promoter DNA methylation status in Tg.AC mice (Cannon et al. 1998). These results underscore that both epigenetic and non-epigenetic regulatory mechanisms must be explored as potential mechanisms of iAs-induced changes in gene expression, and that it must be taken into account that experimental outcomes depend on a variety of factors including differences in experimental design and methods used.

*Immunomodulatory effects.* There is considerable evidence from *in vitro* studies, a range of animal models, and chronically/prenatally exposed human populations that iAs has various immunomodulatory effects (reviewed by Dangleben et al. 2013). Interestingly, the effects of iAs on the immune system are complex and seemingly divergent as they include evidence of both immunosuppression and inflammation, and markers of both effects have been observed simultaneously in exposed populations (Soto-Peña et al. 2006). At the molecular level, there are several indications that the immune system is a major target of iAs toxicity as immune response is a predominant function associated with perturbed mRNAs, miRNAs, and proteins in the cord blood of iAs-exposed newborns from Mexico or Thailand (Ahmed et al. 2011; Bailey et al. 2014; Fry et al. 2007; Rager et al. 2014). As discussed below, the immunosuppressive and proinflammatory effects may contribute not only to long-term health effects of iAs such as cancers but to adverse short-term health effects associated with early-life iAs exposure as well.

Immunosuppression is a significant health concern because of the major roles of the immune system in functions such as healing wounds and providing defense against microbial infections and cancers (Reuter et al. 2010; Vesely et al. 2011). Indicators of immunosuppression that have been observed in prenatally exposed infants in Bangladesh include higher mortality, reduced thymic indices, reduced T-cell function, and higher morbidity rates from pneumonia and diarrhea (Nadeau et al. 2014; Rahman A et al. 2010, 2011; Raqib et al. 2009). There is evidence that more moderate levels of prenatal exposure can also affect immune response, as prenatal iAs exposure was associated with total number of infections requiring physician visit/prescription medication in infants in New Hampshire (Farzan et al. 2013b). It is currently unknown whether immunosuppressive effects associated with prenatal iAs exposure persist into later life. If these effects are sustained, they will likely increase the susceptibility to infections and chronic diseases including cancers that have been associated with iAs exposure (DeWitt et al. 2012; Vega et al. 2009).

There are also indicators of proinflammatory effects in prenatally exposed infants such as the increased expression of proinflammatory genes at the mRNA and protein levels in newborn cord blood, evidence of oxidative damage in the placenta, and increased expression of proinflammatory proteins in the placenta such as IL1-β, TNF, and IFN-γ (Ahmed et al. 2011; Bailey et al. 2014; Fry et al. 2007; Nadeau et al. 2014). As a potent producer of reactive oxygen species (ROS), iAs can stimulate proinflammatory effects that not only cause macromolecule damage but also activate ROS-sensitive signaling pathways implicated in disease development such as those that involve mitogen-activated protein kinases (MAPKs) and the transcription factor nuclear factor kappa-light-chain-enhancer of activated B cells (NF-κB) (Sesti et al. 2012). Of note, inflammation is associated with several diseases associated with iAs exposure including adverse birth/pregnancy outcomes, and a sustained inflammatory state is implicated in the development of several chronic diseases associated with iAs exposure including cancers, diabetes mellitus, atherosclerosis, and liver fibrosis (Challis et al. 2009; Cosentino and Egidy Assenza 2004; Iredale 2007; Libby et al. 2002; Vahter 2007; Valko et al. 2006). Therefore, it is likely that susceptibility to early- and later-life disease development may be linked in part to the complex impacts of prenatal iAs exposure on the immune system.

## Conclusions

Studies in both animal models and human populations have revealed that exposure to iAs during key developmental periods is associated with the development of latent health effects including cancers. Because of the latent nature of many of the effects of iAs exposure, the actual extent of the impact of such exposure is likely underestimated. Although the research relating early-life exposure to iAs and the development of long-term health effects continues to advance, many knowledge gaps remain. Detailed molecular analyses in animal models that employ gene knockouts or chemical inhibition of targeted pathways are required to elucidate the precise mechanisms linking iAs exposure and adverse health effects. In addition, relationships must be identified between early-life iAs exposure, molecular alterations, and short-term and long-term health impacts. In many cases, it is unknown whether molecular alterations associated with iAs exposure are persistent. Thus future research should help to differentiate between adaptive versus adverse alterations and may establish biomarkers of exposure and disease susceptibility. It is also clear that some disease-specific mechanisms such as CSCs exist, but that others such as epigenetic reprogramming and alterations in immune function may contribute to the development of numerous adverse health impacts. Studies must also continue to examine effects observed across a wide range of iAs exposure levels in different populations across the globe, which will help inform dose–response relationships, sex-dependent impacts, and cofactors contributing to disease susceptibility. Taken together, the studies described here underscore that there are likely multiple mechanisms that contribute to the development of latent diseases associated with early-life iAs exposure.
